# People–centred care versus clinic–based DOT for continuation phase TB treatment in Armenia: a cluster randomized trial

**DOI:** 10.1186/s12890-020-1141-y

**Published:** 2020-04-25

**Authors:** Vahe Khachadourian, Nune Truzyan, Arusyak Harutyunyan, Varduhi Petrosyan, Hayk Davtyan, Karapet Davtyan, Martin van den Boom, Michael E. Thompson

**Affiliations:** 1 Avedisian Onanian Centre for Health Services Research & Development, Gerald and Patricia Turpanjian School of Public Health, American University of Armenia, Yerevan, Armenia; 2 Department of Epidemiology, Fielding School of Public Health, University of California, Los Angeles, Los Angeles, USA; 3 National Tuberculosis Control Centre, Ministry of Health of the Republic of Armenia, Yerevan, Armenia; 4 Joint Tuberculosis, HIV & Viral Hepatitis Programme, Division of Health Emergencies and Communicable Diseases, WHO/Europe, Copenhagen, Denmark; 5 Department of Public Health Sciences, University of North Carolina at Charlotte, Charlotte, USA

**Keywords:** Patient-Centreed care, Tuberculosis, Counselling

## Abstract

**Background:**

WHO’s directly observed therapy (DOT) strategy for tuberculosis (TB) treatment depends upon a well–organized healthcare system. This study sought to evaluate the effectiveness of self-administered drug intake supported by a family member versus in-clinic DOT.

**Methods:**

This open–label, nationally-representative stratified cluster randomized controlled non–inferiority trial with two parallel equal arms involved drug–susceptible pulmonary TB patients in the continuation treatment phase. We randomly assigned outpatient–TB–centres (52 clusters) to intervention and control arms. The intervention included an educational/counseling session to enhance treatment adherence; weekly visits to outpatient–TB–centres to receive medication, and daily SMS medication reminders and phone calls to track adherence and record side effects. Controls followed clinical DOT at Outpatient–TB–centres. Both groups participated in baseline and 4–5 months follow–up surveys. The trial’s non–inferiority comparisons include: treatment success as the clinical (primary) outcome and medication adherence (self–reported), knowledge, depressive symptoms, stigma, quality of life, and social support as non–clinical (secondary) outcomes.

**Results:**

Per–protocol analysis showed that the intervention (*n* = 187) and control (*n* = 198) arms achieved successful treatment outcome of 92.0 and 92.9%, respectively, indicating that the treatment success in the intervention group was non–inferior to DOT. Knowledge, depression, stigma, quality of life, and social support also showed non–inferiority, demonstrating substantial improvement over time for knowledge (change in the intervention = 1.05: 95%CL (0.49, 1.60); change in the control = 1.09: 95%CL (0.56, 1.64)), depression score (change in the intervention = − 3.56: 95%CL (− 4.99, − 2.13); change in the control = − 1.88: 95% CL (− 3.26, − 0.49)) and quality of life (change in the intervention = 5.01: 95%CL (− 0.64, 10.66); change in the control = 7.29: 95%CL (1.77, 12.81)). The intervention resulted in improved treatment adherence.

**Conclusions:**

This socially empowering alternative strategy might be a preferable alternative to DOT available to patients in Armenia and in other countries. Further research evaluating cost effectiveness of the intervention and generalizability of the results is warranted.

**Trial registration:**

Clinicaltrials.gov: NCT02082340, March 10, 2014.

## Background

Despite concerted efforts, tuberculosis (TB) remains one of the world’s leading infectious diseases. Promisingly, 97% of all TB diagnoses in 2015 were drug–susceptible [1]. Unfortunately, nearly half fail to adhere to their treatment, often leading to drug resistance (DR) and death [2, 3]. Lack of knowledge, dissatisfaction with healthcare services, lack of social support, and depression contribute to non-adherence [4–7].

Although most healthcare systems follow the World Health Organization (WHO) recommended directly observed therapy (DOT), [8] the rate of relapsed cases developing Multi-Drug Resistant (MDR) TB remains high. In former Soviet countries, the MDR–TB rate is typically 20% higher than the global average, but in Armenia, it is twice that: 47% [1].

Generally, DOT is not superior to self–administered treatment [3]; thus, for drug susceptible patients, alternative TB treatment strategies might be effective in improving adherence while also providing additional benefits to the patient and the healthcare delivery system [7, 9]. For example, strategies that emphasize family members and community [7, 9–12] enhance the effectiveness of medical efforts, improve patients’ quality of life, [13] and contribute to the detection, referral and treatment of people with TB [14]. Using mobile phone text messages (SMS) to remind patients with chronic conditions, enhances treatment adherence and reduces transportation costs [15]. Although several studies have evaluated SMS messaging’s effectiveness for TB treatment [16–20], the body of evidence remains limited and the need for further studies is warranted.

TB treatment adherence is not due to a single factor, but to a coordinated combination of strategies that target patients’ knowledge, emotional status, treatment–related expenses, and convenience through patient–centred care that concurrently enhances patient rights and equities [1]. This randomized controlled trial evaluated the effectiveness of a multi–component TB care strategy (self-administered drug intake with the support of a family member, TB counselling, calls, and SMS text reminders) versus DOT in terms of treatment outcomes (primary, clinical outcome) among drug–susceptible pulmonary TB patients. The study additionally assessed adherence to treatment protocol, TB knowledge, depressive symptoms, stigma, quality of life, and social support (secondary, non–clinical outcomes) .

## Methods

### Study design

The Innovative Approach to Tuberculosis Care in Armenia (IATCA) was an open–label stratified cluster randomized controlled non–inferiority trial with two parallel equal arms (intervention and control). It targeted the Continuation Phase of TB Treatment (CPT) and was implemented through Armenia’s TB outpatient centres (TBOC). We conducted the trial in accordance with good clinical practice guidelines and the Declaration of Helsinki. The American University of Armenia Institutional Review Board (IRB) reviewed and approved the study protocol. This study is registered with ClinicalTrials.gov (NCT: 02082340) and its methods are described in greater detail elsewhere [21].

### Randomization and masking

The study included all TBOCs in Armenia that had more than five drug susceptible TB patients in 2012 (52 TBOCs out of the 60 total TBOCs). We stratified the centres by patient load (low [up to 10 patient per year], average [11 to 25 patients per year], high [more than 25 patients per year]) and treatment success [22] (up to 64%; from 65 to74%; above 74%) yielding nine distinctive categories. Computer assisted block randomization resulted in 26 centres in each arm with intracluster correlation equal to 0.04 and an estimated design effect of 1.6 [21]. Stratifying TBOCs by patient load also improved the balance between the number of physicians and nurses in the clinics across the study arms, which varied between 2 health care workers in TBOCs with a small patient load to 7 health care workers in TBOCs with a high patient load. Interviewers assessing non–clinical outcomes were not blinded at baseline but were at follow-up. Independent of the research team, each TBOC physician assessed and reported treatment outcome to the National Tuberculosis Control Centre (NTCC).

### Participants and sample size

Study participants were drug susceptible pulmonary TB patients starting their CPT TB treatment in a selected TBOC between March and December 2014, at least 18 years old at enrolment, and able to communicate in Armenian. A patient’s residence determined assignment to a specific TBOC. The corresponding TBOC physician and NTCC psychologist verified the eligibility of each patient.

We conservatively calculated the sample size needed to conduct a superiority trial. We assumed a 10% difference in the primary outcome, [23] an alpha of 0.05 and power of 80%. Considering the potential for losses to follow-up, we used a design effect of two (rather than 1.6); thus, requiring 190 for each arm.

### Procedures

Where available, intervention arm patients identified family supporters who participated in a counselling session and supported them during their treatment period. Trained interventionists (a psychologist and a nurse) conducted a single educational and psychological counselling and empowerment session for the TB patient and, as available, his/her supportive family member, and other interested family members. Session topics included: TB symptoms, route of transmission, treatment strategies, the importance of treatment adherence, prevention and infection control, addressing TB–related stigma and common myths, handling side effects, and a detailed explanation of the treatment protocol. The session lasted 120 min on average, exceeding the initially estimated duration by 30 min. The longer duration of the session was due to participants’ significant interest in the topics covered as well as an extended question and answer period at the end of the session. After the session, patients began self–administered drug–intake supervised by a trained family member. Once per week, patients visited the TBOC healthcare provider to receive the next week’s medication in a pill–minder box. The research team sent daily morning SMS messages to intervention patients. The team placed daily phone calls to supporting family members to inquire about patients’ adherence to the prescribed treatment as well as any potential side effects. Patients lacking a supporting family member were responsible for all activities defined for the supportive family member.

Control arm patients received DOT during their continuation phase treatment, in accord with Armenia’s national strategy (comparable to implementations of WHO TB treatment guidelines worldwide).

### Outcomes

The primary outcome was treatment success at the end of the continuation phase. Following WHO conventions, we considered “cured” or “treatment completed” a “treatment success.” [22] WHO Definitions and reporting framework for tuberculosis [22] are presented in the supplemental material. In both intervention and control arms, the TBOC treating physician clinically assessed and reported the outcome independent of the research team.

We utilized face–to–face surveys administered by trained research staff to collect non–clinical (secondary) outcome data: self–reported (patient–reported) adherence to TB treatment, knowledge, depressive symptoms, stigma, quality of life, and social support [24–27]. We measured all non–clinical outcomes (except self–reported adherence) at baseline and follow–up. We measured treatment adherence by self–reported (patient–reported) and family member–reported frequency of receiving and taking TB medications. We adapted the knowledge variable from previous work [24, 25], with a score range of 0 to 31 (see the Additional file 1). We assessed depression symptoms using a validated modified Armenian version of the Centre for Epidemiological Studies Depression (CES–D) scale which had a score range of 0 to 60, with higher scores indicating higher levels of depression symptoms [26]. We modified the Van Rie stigma scale to measure stigma [28]. The original scale measures stigma of tuberculosis from the community perspectives. We modified the questions to measure the behaviour of family members toward the tuberculosis patient from patients’ perspective. Its score ranged from 0 to 27 (see the Additional file 1). We measured quality of life using the EQ-5D [29]. The responses to the quality of life scales yield an index utility score anchored at 0 for death and 1 for perfect health. The scores were multiplied by 100 to increase precision of the reported estimates. We used the Berlin social support scale to measure social support of family members toward TB patients. Scores ranged from 0 to 45 [30].

### Statistical analyses

We used SAS 9.4 statistical package for our analyses. We included only complete cases (those without a missing value for the outcome under analysis). We assessed the distribution of socio–demographic characteristics and other potential confounding variables across the two arms using frequency and proportion for categorical variables, and mean and standard deviation for continuous variables. We analysed the clinical outcome using a Generalized Estimating Equations (GEE) binomial regression with identity link, considering TB clinics as the clusters following the intention to treat and per protocol principles [31]. We used an exchangeable correlation structure to account for the correlation between treatment outcomes within the clusters [32].

Following per–protocol analysis principle, linear mixed effect models including random effect for TB clinics and subjects (random intercepts) tested the non–clinical outcomes. For each non–clinical outcome, we adjusted for baseline values and the change over time. The final results present the change in the outcome over the study period (from baseline to follow-up) in each arm, adjusted for the baseline measures [33].

We determined absolute differences for outcomes based on efficacy established in other reported TB non–inferiority or equivalence trials [34] and our previous research experience [24, 26, 27]. We obtained the non–inferiority margin of − 3% (i.e. − 0.03) on the absolute difference scale for the clinical outcome (treatment success). We declared non–inferiority if the lower limit of the 95% CI was greater than the corresponding non–inferiority margin for the positive outcomes (i.e., treatment success, knowledge score, social support, and quality of life scores). For the negative outcomes (i.e., stigma score, depression score), we declared non–inferiority if the upper limit of the 95% CI was smaller than the corresponding non–inferiority margin. Similarly, we defined non–inferiority margins for knowledge and social support to be 1.5, and for stigma to be − 1.5. We applied non–inferiority margins of 2.0 for depression scores, and 15 for quality of life scores.

We conducted sensitivity analyses using multiple imputation and last observation carried forward to assess the potential impact of missing data in the non-clinical outcomes (see Additional file 1 for details). We reported our study using the Consolidated Standards of Reporting Trials (CONSORT) guidelines.

## Results

**Fig. 1:**
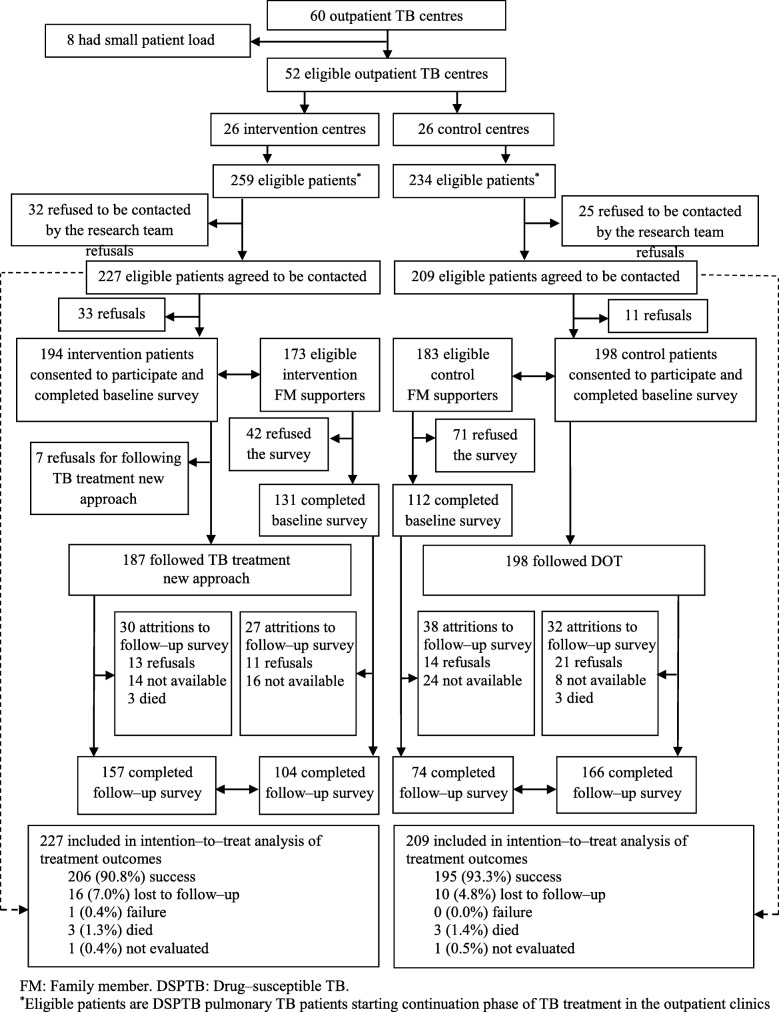
Trial profile

Between 12 March and 26 December 2014, we identified and contacted 259 eligible TB patients from intervention and 234 TB patients from control TBOCs. Of the eligible patients, 227 and 209 from the intervention and control arms agreed to be contacted by the research team. A total of 194 patients from the intervention TBOCs and a total of 198 patients from the control TBOCs consented to participate in the study and completed the baseline survey (Fig. 1). Data on treatment outcomes were available for all the patients who agreed to be contacted by the research team (227 intervention and 209 control TB patients), regardless of whether they consented to participate in the study or not.

CPT averaged 4.2 months and was equal in both arms. Table 1 presents participants’ general socio–demographic characteristics and demonstrates that the randomization process achieved comparable groups. Table 2 details patients’ primary outcome at follow–up according to the intent to treat and per–protocol analyses principles. According to per–protocol analysis, at treatment completion, the intervention arm yielded a 92.0% “treatment success” rate*,* (including 32 (17.1%) “cured” plus 140 (74.9%) “treatment complete”), non–inferior to the control arm’s 92.9% “treatment success” rate (including 26 (13.1%) “cured” plus 158 (79.8%) “treatment complete”). The intention to treat analysis yielded results consistent with the per–protocol analysis. Table 3 presents the non-clinical outcomes (secondary outcomes) according to the per-protocol analyses principles.

**Table 1:** Baseline characteristics of the study population (patients)

Variable	Intervention (***N*** = 187)		Control (***N*** = 198)	
Age (years), mean (SD)	45.2	(15.7)	47.8	(14.9)
Gender (males), *n* (%)	148	(79.1%)	150	(75.8%)
Married, *n* (%)	136	(72.7%)	145	(73.2%)
Education > 10 years	75	(40.3%)	76	(38.4%)
Alcohol abuse, *n* (%)	56	(30.4%)	54	(27.6%)
Current smokers, *n* (%)	102	(54.6%)	104	(52.8%)
Ever being a migrant labourer, *n* (%)	75	(41.4%)	84	(43.1%)
Employed, *n* (%)	47	(26.0%)	49	(24.9%)
Wealth score, mean (SD)	4.0	(1.8)	4.1	(1.9)
Have no FM supporter, *n* (%)	14	(7.5%)	15	(7.6%)
Knowledge score, mean (SD)	22.9	(3.3)	22.0	(3.8)
Depression score, mean (SD)	7.8	(9.7)	6.5	(8.8)
Stigma score, mean (SD)	0.4	(1.6)	0.9	(3.2)
Quality of life, mean (SD)	73.8	(29.6)	74.2	(27.5)
Support score, mean (SD)	42.0	(5.6)	41.5	(6.9)
Sputum Smear positive, *n* (%)	50	(26.7%)	35	(17.7%)
Number of TB treatments, mean (SD)	1.2	(0.6)	1.3	(0.7)
Retreated, *n* (%)	27	(14.4%)	46	(23.4%)
HIV/AIDS positive, *n* (%)	14	(8.4%)	7	(4.1%)

^*^Data not available for all randomized patients

**Table 2:** Primary outcome of patients at the follow-up according to intention to treat and per-protocol analysis principles

Outcome	Intention to treat analysis		Per–protocol analysis	
	Intervention ***n*** = 227	Control ***n*** = 209	Intervention ***n*** = 187	Control ***n*** = 198
Treatment outcome				
*Success*	206 (90.8%)	195 (93.3%)	172 (92.0%)	184 (92.9%)
*Lost to follow-up*	16 (7.0%)	10 (4.8%)	12 (6.4%)	10 (5.0%)
*Failure*	1 (0.4%)	0 (0.0%)	0 (0.0%)	0 (0.0%)
*Death*	3 (1.3%)	3 (1.4%)	3 (1.6%)	3 (1.5%)
*Not evaluated*	1 (0.4%)	1 (0.5%)	0 (0.0%)	1 (0.5%)

**Table 3:** Secondary outcome of patients at the follow-up according to per-protocol analysis principles

Outcome	Intervention		Control	
	Valid response	Mean (SD)	Valid response	Mean (SD)
Knowledge score	155	23.9 (3.1)	165	23.1 (3.5)
Depression score	152	4.3 (7.5)	162	4.7 (7.6)
Stigma score	155	0.7 (3.1)	158	0.6 (2.7)
Quality of life	149	78.8 (25.9)	157	81.6 (24.5)
Support score	151	43.2 (5.3)	160	41.8 (7.5)

The GEE model accounting for cluster effect by TBOCs, compared the treatment success between the study arms according to the intention to treat as well as per–protocol analysis principles. The analysis did not find any clinically and statistically meaningful difference (Table 4). The analysis documented that 100% of intervention arm patients received their drugs as scheduled, while control arm adherence was substantially lower at 87.3% (*p* < 0.01). Family supporters reported lower adherence: 99.0% for intervention patients and 79.7% (p < 0.01) for controls.

**Table 4:** Regression analysis of treatment success comparison between intervention and control arms

GEE^**a**^	Beta estimate	95% confidence limits		***P***–value
**Treatment success difference (Intervention – Control)**				
Intention to treat analysis	−0.002	−0.018	0.015	0.84
Per-protocol treatment	−0.021	−0.036	−0.006	0.02

^a^Generalized Estimating Equation model (with TB outpatient clinics as clusters)

The average knowledge score had increased notably at follow–up, improving 1.05 in the intervention and 1.09 in the control group. The random intercept model (Table 5) demonstrated no difference in improvement between the arms. The random intercept model demonstrated the mean depressive symptom score substantially decreased across both arms (by 3.56 in the intervention group and 1.88 in the control group), with a slightly larger decline in the intervention group (*p* = 0.10). Comparisons of the outcomes between the intervention and control groups yielded estimates that did not cross their corresponding non–inferiority margins (Table 5).

**Table 5:** Regression analysis of TB patients’ and their family members’ non–clinical outcomes by intervention

Random intercept model^**a**^	TB patients				Family members			
	Beta estimate	95% confidence limits		***P***–value	Beta estimate	95% confidence limits		***P***–value
**Knowledge score change**^**b**^								
Intervention	1.05	0.49	1.60		2.02	1.30	2.75	
Control	1.09	0.56	1.64		1.43	0.60	2.27	
Difference (Intervention – Control)	−0.05	−0.82	0.73	0.73	0.59	−0.52	1.70	0.29
**Depression score change**^**b**^								
Intervention	−3.56	−4.99	−2.13		− 3.44	−4.96	−1.92	
Control	−1.88	−3.26	−0.49		− 1.78	−3.53	−0.02	
Difference (Intervention – Control)	−1.68	−3.67	0.30	0.10	− 1.66	−3.98	0.66	0.16
**Stigma score change**^**b**^								
Intervention	0.23	−0.33	0.80		− 0.73	−1.25	−0.22	
Control	−0.28	−0.84	0.27		− 0.99	−1.59	−0.39	
Difference (Intervention – Control)	0.52	−0.27	1.31	0.20	0.26	− 0.53	1.05	0.52
**Support score change**^**b**^								
Intervention	1.02	−0.25	2.28		−0.04	−0.97	0.90	
Control	0.06	−1.15	1.27		− 0.43	−1.50	0.64	
Difference (Intervention – Control)	0.95	−0.80	2.70	0.28	0.40	−1.03	1.82	0.58
**Quality of life score change**^**b**^								
Intervention	5.01	−0.64	10.66					
Control	7.29	1.77	12.81					
Difference (Intervention – Control)	−2.28	10.18	5.61	0.57				

^a^Mixed effect models including random effect for TB clinics and subjects (random intercepts)

^b^Change is the difference in the outcome measure at the follow-up and baseline

The random intercept model demonstrated that the mean depression score from baseline to follow-up substantially decreased for patients’ family supporters in both arms (intervention = − 3.44: 95%CL (− 4.96, − 1.92); control = − 1.78: 95%CL (− 3.53, − 0.02)).The larger decline among the intervention arm was not substantially different from the control arm (*p* = 0.16) (Table 5). The stigma score among patients’ family supporters also decreased in both arms while social support did not notably change over time and were comparable across study arms.

Most intervention patients reported that the text messages were helpful in reminding them to take their drugs everyday (78.4%) and on time (66.2%), and to visit the TBOC weekly (58.3%). About 10% reported that the messages did not change their behaviour, because they would have taken the drugs regardless of those reminders. Most (80.9%), family supporters reported that the phone calls helped them feel confident that the disease was under control.

Our sensitivity analyses (further details in the Additional file 1), using multiple imputation and last observation carried forward strategies for the missing values for the secondary outcomes at follow-up, showed the robustness of our findings, enhancing their credibility independent of our analytic assumptions.

## Discussion

This randomized trial evaluated effectiveness of a people-centred TB care, a strategy that is well aligned with and supported by the people-centred model of care advocated by the WHO [35]. The results showed that the intervention yielded clinical outcomes (treatment success) non–inferior to regular DOT (above 90% in both groups). While not demonstrating superiority, as with a cluster randomized controlled trial in Nepal [7] and a retrospective cohort study in Pakistan, [36] our findings are consistent with a Cochrane systematic review showing equal success for DOT and self–administered strategies [10].

We designed the intervention strategy to increase adherence through education and psychological support, thereby reducing losses to follow–up and increasing completion success. The intervention might have had less impact on treatment success rates than anticipated because the majority losses to follow–up occurred before the continuation phase. By planning the intervention for the CPT phase, we missed the large group of patients who probably needed more support to continue treatment. Retargeting this intervention to patients during the intensive treatment phase might lead to better overall treatment outcomes through reductions in losses to follow–up and higher adherence rates among those entering CPT.

A 2012 study conducted in Armenia documented that, at most, 32% of drug susceptible TB patients reported adherence to DOT and 53% reported self–administered daily intake of medication [37]. Nevertheless, in this trial, 87% of controls reported adherence to DOT; this number was 80% according to the family–members. We observed a similar trend in the treatment success rates. In 2012 and 2013, the two years prior to the study, the TB treatment success rates among those who initiated the continuation treatment phase were 82.4 and 86.2%, respectively. Similarly, the overall treatment success rates in 2015 and 2016, the two years following the clinical trial, were 81.9 and 81.8% (Table 6). Nevertheless, during this study period, treatment success rates among patients in the intervention and control arms (above 90%) were notably higher than those observed in the surrounding years. The most plausible explanation for the observed differences is that the treatment success rates in this clinical trial provide better estimations for the efficacy of the treatment strategies rather than their effectiveness [38]. Indeed, the success rate of 92.9% observed in the control arm was consistent with the efficacy of DOT strategy evaluated in other studies [39, 40]. Given the flexible nature of our intervention and lower administrative and logistical requirements relative to DOT, we believe that the gap between effectiveness and efficacy for our innovative treatment would be smaller than for DOT.

**Table 6:** Treatment outcome of TB patients in the clinical trial, during two years prior to the clinical trial, and during the two years following the clinical trial

Treatment outcome	Year					
	2012^a^ ***n*** (%)	2013^a^ ***n*** (%)	2014, intervention ***n*** (%)	2014, control ***n*** (%)	2015^a^ ***n*** (%)	2016^a^ ***n*** (%)
Success	511 (82.4%)	513 (86.2%)	206 (90.8%)	195 (93.3%)	421 (81.9%)	310 (81.8%)
Lost to follow-up	78 (12.6%)	53 (8.9%)	16 (7.0%)	10 (4.8%)	65 (12.6%)	46 (12.1%)
Failure	6 (1.0%)	9 (1.5%)	1 (0.4%)	0 (0.0%)	7 (1.4%)	6 (1.6%)
Death	22 (3.5%)	16 (2.7%)	3 (1.3%)	3 (1.4%)	21 (4.1%)	9 (2.4%)
Not evaluated	3 (0.5%)	4 (0.7%)	1 (0.4%)	1 (0.5%)	0 (0.0%)	8 (2.1%)

^a^Among TB patients corresponding to the same eligibility criteria applied in the clinical trial (Drug sensitive pulmonary TB patients starting continuation phase of TB treatment in the outpatient clinics)

At follow-up, patients from both arms demonstrated substantial increase in TB knowledge, supporting the findings of other international studies [10, 11] and the 2012 Armenia pilot study [24]. One explanation for these unexpectedly high adherence rate and knowledge gains observed in the control group is compensatory rivalry from the control cluster TB physicians. As Mitchell and Selmes describe, physicians often fail to explain the benefits and side effects of a medication adequately. The control TBOC physicians might have changed their behaviour, improving their DOT and patient counselling given they were aware of the trial and its potential ramifications for their practices. Lack of national annual surveillance data on TB patients’ knowledge, limits our drawing of any firm conclusions. Empowering patients and their family supporters through counselling positively affects the adherence and treatment process. Moreover, involving family members extends the people–centred approach described by Mezzich et al. [41]

Intervention patients showed substantially greater decreases in depressive symptomatology. This finding demonstrates the importance of psychological counselling during TB treatment and supports other studies’ findings that patients are less likely to become depressed and are more adherent when they have strong support [7, 9, 12, 42] and that strong support can enhance immune function [43]. Close family ties and strong family support are common and highly valued in Armenia [44]. That support provides a strong foundation for the success of this intervention.

## Conclusions

A more people–centred treatment approach to TB that includes educational, psychological, and family support components might be a preferable alternative [to DOT] available to patients in Armenia and in other countries. It can be less expensive, more flexible and non-inferiorly effective as DOT. Further research on the impact as well as relative cost effectiveness of similar people–centred interventions for TB patients in other countries is warranted. Future studies might also consider initiating the intervention during the intensive phase of TB treatment as it could result in much better adherence rates throughout the full course of TB treatment and lead to even better clinical outcomes.

## Supplementary information

**Additional file 1 **Treatment outcome definition and measurement of secondary outcomes. **Table S1.** Regression analysis of TB patients’ non-clinical outcomes by intervention (multiple imputation of all missing values) **Table S2.** Regression analysis of TB patients’ non-clinical outcomes by intervention (last observation carried forward strategy)

## Data Availability

For access to data, please contact the corresponding author.

## Abbreviations

CES–D: The Centre for Epidemiological Studies Depression
CPT: Continuation Phase of TB Treatment
DOT: Directly Observed Treatment
DR: Drug Resistance
GEE: Generalized Estimating Equations
IATCA: The Innovative Approach to Tuberculosis Care in Armenia
IRB: Institutional Review Board
NTCC: The National Tuberculosis Control Centre
MDR: Multi Drug Resistance
SMS: Mobile phone text messages
TB: Tuberculosis
TBOC: TB Outpatient Centres
WHO: World Health Organization
