# Supplementary material for: People–centred care versus clinic–based DOT for continuation phase TB treatment in Armenia: a cluster randomized trial
Source: BMC Pulm Med. 2020 Apr 25;20:105. doi: 10.1186/s12890-020-1141-y (PMC7183136; doi:10.1186/s12890-020-1141-y)
Supplement: Supplementary file 1 — Additional file 1 Treatment outcome definition and measurement of secondary outcomes. Table S1. Regression analysis of TB patients’ non-clinical outcomes by intervention (multiple imputation of all missing values) Table S2. Regression analysis of TB patients’ non-clinical outcomes by intervention (last observation carried forward strategy) [file 12890_2020_1141_MOESM1_ESM.docx]

**Appendix**

**Treatment outcome definition**

Treatment outcomes for TB patients (excluding patients treated for Multidrug- and rifampicin-resistant tuberculosis (RR-TB or MDR-TB)) were evaluated based on the World Health Organization (WHO) treatment outcome definitions^[[1]](#footnote-1)^ as presented below.

*Cured*: “A pulmonary TB patient with bacteriologically confirmed TB at the beginning of treatment who was smear- or culture-negative in the last month of treatment and on at least one previous occasion”

*Treatment completed*: “A TB patient who completed treatment without evidence of failure BUT with no record to show that sputum smear or culture results in the last month of treatment and on at least one previous occasion were negative, either because tests were not done or because results are unavailable.”

*Treatment failure*: “A TB patient whose sputum smear or culture is positive at month 5 or later

during treatment.”

*Death*: “A TB patient who dies for any reason before starting or during the course

of treatment.”

*Lost to follow-up*: “A TB patient who did not start treatment or whose treatment was

interrupted for 2 consecutive months or more.”

*Not evaluated*: “A TB patient for whom no treatment outcome is assigned. This includes

cases “transferred out” to another treatment unit as well as cases for whom the treatment outcome is unknown to the reporting unit.”

*Treatment success*: “The sum of cured and treatment completed.”

**Questionnaires**

Knowledge questionnaire

| **Please answer the following questions.** | | |
| --- | --- | --- |
|  | How can a person get infected with TB?  Choose all the answers that you think are correct. | 1. By being in contact with TB patients 2. By greeting a TB patient with a handshake 3. Through the air when a person with TB coughs or sneezes 4. By sharing the same platter 5. By sharing the same sanitary utensils 6. By touching various objects in public places (doorknobs, handles in transport, etc.) 7. Other ___________________   **88**. Do not know/ Difficult to answer |
|  | What are the signs of pulmonary TB?  Choose all the answers that you think are correct. | 1. Cough, lasting longer than 3 weeks 2. Bloody cough 3. Rash 4. Weight loss 5. Elevated temperature 6. Nausea 7. Severe headache 8. Chest pain 9. Visual disturbance 10. Difficulty breathing/not enough air 11. Weakness 12. Night sweating 13. Other _______________________________   **88**. Do not know/ Difficult to answer |
|  | How can a person in the infective stage of TB prevent the spread of disease?  Choose all the answers that you think are correct. | 1. Immediately begin treatment 2. Avoid shaking hands 3. Cover mouth and nose when coughing or sneezing 4. Wash hands frequently 5. Ventilate the room frequently 6. Wear a mask 7. Try interacting with people in confined areas 8. Avoid using public transportation 9. Other (note): ____________________________   **88.** Do not know/ Difficult to answer |
|  | What is the best method of TB treatment?  Please select one best method. | 1. Home remedies, eg. Herbal medication, good nutrition, etc 2. Resting at home, without using any medication 3. Treatment with medication purchased from pharmacies 4. Taking drugs as prescribed by the doctor 5. Other_________________________________ |
|  | What can happen if the tuberculosis patient does not follow their treatment scheme correctly?  Choose all the answers that you think are correct. | 1. Will remain sick for a longer period 2. Will spread tuberculosis to other people 3. Will develop drug-resistant tuberculosis 4. There won’t be any significant changes 5. Other_________________________________ |

Stigma scale for TB patients

|  | **The following questions are about your family perspectives toward tuberculosis**  Please choose to which extent you agree or disagree with each particular statement bellow | **Strongly**  **Disagree** | **Somewhat**  **Disagree** | **Somewhat**  **Agree** | **Strongly**  **Agree** |
| --- | --- | --- | --- | --- | --- |
| 1 | Some of your family members may not want to eat or drink with you. | 􀀀 0 | 􀀀 1 | 􀀀 2 | 􀀀 3 |
| 2 | Some of your family members feel uncomfortable about being near you. | 􀀀 0 | 􀀀 1 | 􀀀 2 | 􀀀 3 |
| 3 | Some of your family members will behave differently towards you for the rest of life. | 􀀀 0 | 􀀀 1 | 􀀀 2 | 􀀀 3 |
| 4 | Some of your family members do not want you to play with children. | 􀀀 0 | 􀀀 1 | 􀀀 2 | 􀀀 3 |
| 5 | Some of your family members keep their distance from you. | 􀀀 0 | 􀀀 1 | 􀀀 2 | 􀀀 3 |
| 6 | Some of your family members do not want to talk to you. | 􀀀 0 | 􀀀 1 | 􀀀 2 | 􀀀 3 |
| 7 | Some of your family members are afraid of you. | 􀀀 0 | 􀀀 1 | 􀀀 2 | 􀀀 3 |
| 8 | Some of your family members try not to touch you. | 􀀀 0 | 􀀀 1 | 􀀀 2 | 􀀀 3 |
| 9 | Some of your family members prefer not to live with you. | 􀀀 0 | 􀀀 1 | 􀀀 2 | 􀀀 3 |

Stigma scale for family supporters

|  | **The following questions are about your perspectives toward the family member with TB**  Please choose to which extent you agree or disagree with each particular statement bellow | **Strongly**  **Disagree** | **Somewhat**  **Disagree** | **Somewhat**  **Agree** | **Strongly**  **Agree** |
| --- | --- | --- | --- | --- | --- |
| 1 | I do not want to eat or drink with my family member who has TB. | 􀀀 0 | 􀀀 1 | 􀀀 2 | 􀀀 3 |
| 2 | I feel uncomfortable about being near my family member who has TB. | 􀀀 0 | 􀀀 1 | 􀀀 2 | 􀀀 3 |
| 3 | I will behave differently towards my family member who has TB for the rest of life. | 􀀀 0 | 􀀀 1 | 􀀀 2 | 􀀀 3 |
| 4 | I do not want my family member who has TB to play with children. | 􀀀 0 | 􀀀 1 | 􀀀 2 | 􀀀 3 |
| 5 | I keep the distance from my family member who has TB. | 􀀀 0 | 􀀀 1 | 􀀀 2 | 􀀀 3 |
| 6 | I do not want to talk to my family member who has TB. | 􀀀 0 | 􀀀 1 | 􀀀 2 | 􀀀 3 |
| 7 | I am afraid of my family member who has TB. | 􀀀 0 | 􀀀 1 | 􀀀 2 | 􀀀 3 |
| 8 | I try not to touch my family member who has TB. | 􀀀 0 | 􀀀 1 | 􀀀 2 | 􀀀 3 |
| 9 | Some of your family members prefer not to live with you. | 􀀀 0 | 􀀀 1 | 􀀀 2 | 􀀀 3 |

**Sensitivity analyses**

Assuming multivariate normal distribution, we used Markov Chain Monte Carlo (MCMC) simulation to perform the multiple imputation (n = 40) for all the missing outcomes for the secondary outcomes. We further repeated our analysis using the baseline values of the secondary outcomes to replace the missing data for the outcomes at the follow–up (last observation carried forward).

**Table S1. Regression analysis of TB patients’ non-clinical outcomes by intervention (multiple imputation of all missing values)**

| **Random intercept model^a^** | **TB patients** | | | |  |  |
| --- | --- | --- | --- | --- | --- | --- |
|  | **Beta estimate** | **95% confidence limits** | | **P-value** |  |  |
| **Knowledge score change^b^** | | | | |  |  |
| Intervention | 1.11 | 0.51 | 1.71 |  |  |  |
| Control | 1.24 | 0.62 | 1.86 |  |  |  |
| Difference (Intervention – Control) | –0.13 | –0.91 | 0.64 | 0.74 |  |  |
| **Depression score change^b^** | | | | |  |  |
| Intervention | –3.57 | –5.11 | –2.03 |  |  |  |
| Control | –1.97 | –3.52 | –0.43 |  |  |  |
| Difference (Intervention – Control) | –1.60 | –3.51 | 0.32 | 0.10 |  |  |
| **Stigma score change^b^** | | | | |  |  |
| Intervention | 0.30 | –0.39 | 0.99 |  |  |  |
| Control | –0.26 | –1.00 | 0.47 |  |  |  |
| Difference (Intervention – Control) | 0.58 | –0.27 | 1.43 | 0.18 |  |  |
| **Support score change^b^** | | | | |  |  |
| Intervention | 1.08 | –1.00 | 3.16 |  |  |  |
| Control | 0.69 | –1.65 | 3.03 |  |  |  |
| Difference (Intervention – Control) | 0.39 | –1.58 | 2.40 | 0.70 |  |  |
| **Quality of life score change^b^** | | | | |  |  |
| Intervention | 4.37 | –1.08 | 12.43 |  |  |  |
| Control | 5.37 | –0.47 | 11.21 |  |  |  |
| Difference (Intervention – Control) | –1.00 | –8.73 | 6.71 | 0.80 |  |  |
| ^a^ Mixed effect models including random effect for TB clinics and subjects (random intercepts).  ^b^ Change is the difference in the outcome measure at the follow-up and baseline | | | | | | |

**Table S2. Regression analysis of TB patients’ non-clinical outcomes by intervention (last observation carried forward strategy)**

| **Random intercept model^a^** | **TB patients** | | | |
| --- | --- | --- | --- | --- |
|  | **Beta estimate** | **95% confidence limits** | | **P-value** |
| **Knowledge score change^b^** | | | | |
| Intervention | 0.92 | 0.44 | 1.40 |  |
| Control | 0.86 | 0.40 | 1.33 |  |
| Difference (Intervention – Control) | 0.06 | –0.61 | 0.72 | 0.87 |
| **Depression score change^b^** | | | | |
| Intervention | –2.97 | –4.18 | –1.76 |  |
| Control | –1.67 | –2.85 | –0.50 |  |
| Difference (Intervention – Control) | –1.30 | –2.98 | 0.38 | 0.13 |
| **Stigma score change^b^** | | | | |
| Intervention | 0.07 | –0.40 | 0.53 |  |
| Control | –0.30 | –0.74 | 0.15 |  |
| Difference (Intervention – Control) | 0.36 | –0.28 | 1.01 | 0.27 |
| **Support score change^b^** | | | | |
| Intervention | 0.37 | –0.56 | 1.29 |  |
| Control | –0.49 | –1.39 | 0.40 |  |
| Difference (Intervention – Control) | 0.85 | –0.43 | 2.15 | 0.19 |
| **Quality of life score change^b^** | | | | |
| Intervention | 2.38 | –2.12 | 6.87 |  |
| Control | 5.26 | 0.90 | 9.62 |  |
| Difference (Intervention – Control) | –2.9 | –9.15 | 3.38 | 0.36 |

^a^ Mixed effect models including random effect for TB clinics and subjects (random intercepts).

^b^ Change is the difference in the outcome measure at the follow-up and baseline

1. World Health Organization. Definitions and reporting framework for tuberculosis. WHO; 2014. [↑](#footnote-ref-1)
